# *Eurythenes atacamensis* sp. nov. (Crustacea: Amphipoda) exhibits ontogenetic vertical stratification across abyssal and hadal depths in the Atacama Trench, eastern South Pacific Ocean

**DOI:** 10.1007/s12526-021-01182-z

**Published:** 2021-05-14

**Authors:** Johanna N. J. Weston, Liliana Espinosa-Leal, Jennifer A. Wainwright, Eva C. D. Stewart, Carolina E. González, Thomas D. Linley, William D. K. Reid, Pamela Hidalgo, Marcelo E. Oliva, Osvaldo Ulloa, Frank Wenzhöfer, Ronnie N. Glud, Rubén Escribano, Alan J. Jamieson

**Affiliations:** 1 School of Natural and Environmental Sciences, Newcastle University, Newcastle Upon Tyne, NE1 7RU UK; 2 Programa de Doctorado en Oceanografía, Departamento de Oceanografía, Universidad de Concepción, P.O. Box 160 C, Concepción, Chile; 3 Departamento de Oceanografía and Instituto Milenio de Oceanografía, Universidad de Concepción, P.O. Box 160 C, Concepción, Chile; 4 Life Sciences, The Natural History Museum, Cromwell Road, London, SW7 5BD UK; 5 Instituto de Ciencias Naturales Alexander von Humboldt, Facultad de Ciencias del Mar y Recursos Biológicos, Universidad de Antofagasta, Antofagasta, Chile; 6 HGF-MPG Group for Deep Sea Ecology and Technology, Alfred-Wegener-Institute Helmholtz-Center for Polar and Marine Research, 27570 Bremerhaven, Germany; 7 Max Planck Institute for Marine Microbiology, 28358 Bremen, Germany; 8 Department of Biology, Nordcee and HADAL, University of Southern Denmark, 5230 Odense M, Denmark; 9 Tokyo University of Marine Science and Technology, 4-5-7 Konan, Minato-ku, Tokyo, 108-8477 Japan

**Keywords:** Peru-Chile Trench, New species, Cryptic species, Deep sea, Integrated taxonomy, *Eurythenes* key

## Abstract

*Eurythenes* S.I. Smith in Scudder, 1882 (Crustacea: Amphipoda) are prevalent scavengers of the benthopelagic community from bathyal to hadal depths. While a well-studied genus, molecular systematic studies have uncovered cryptic speciation and multiple undescribed lineages. Here, we apply an integrative taxonomic approach and describe the tenth species, *Eurythenes atacamensis* sp. nov., based on specimens from the 2018 Atacamex and RV Sonne SO261 Expeditions to the southern sector of the Peru-Chile Trench, the Atacama Trench (24–⁠21°S). *Eurythenes atacamensis* sp. nov. is a large species, max. observed length 83.2 mm, possesses diagnostic features, including a short gnathopod 1 palm and a chelate gnathopod 2 palm, and a distinct genetic lineage based on a 16S rRNA and COI phylogeny. This species is a dominant bait-attending fauna with an extensive bathymetric range, spanning from 4974 to 8081 m. The RV Sonne SO261 specimens were recovered along a 10-station transect from abyssal to hadal depths and further examined for demographic and bathymetric-related patterns. Ontogenetic vertical stratification was evident across the trench axis, with only juveniles present at abyssal depths (4974–6025 m). Total length-depth analysis revealed that the size of females was unrelated to depth, whereas juveniles followed a sigmoidal relationship with a step-up in size at depths >7200 m. Thus, these bathymetric trends suggest that juveniles and females employ differing ecological strategies in subduction trench environments. This study highlights that even dominant and ecologically important species are still being discovered within the abyssal and hadal environments. Continued systematic expeditions will lead to an improved understanding of the eco-evolutionary drivers of speciation in the world’s largest ecosystem.

## Introduction

The deep ocean is the Earth’s largest ecosystem, extending from the edge of the continental shelf (200 m) to the bottom of the subduction trenches (~11,000 m; Thiel 2003, Stewart and Jamieson 2019), covering approximately 1.4 × 10^9^ km^3^ (Charette and Smith 2010). Despite the extreme environmental conditions of high pressure, low temperature, and limited food availability (Grassle and Maciolek 1992; Smith et al. 2008; Jamieson et al. 2009), the deep ocean harbours a wide range of adapted species (Belyaev 1989; Ebbe et al. 2010). The Amphipoda genus *Eurythenes* S.I. Smith in Scudder, 1882 are prevalent members of the deep ocean, benthopelagic community (Stoddart and Lowry 2004; Havermans 2016). This genus inhabits every ocean across an extensive bathymetric range—observed in polar waters (Ainley et al. 1986; Bowman and Manning 1972), on the abyssal plains (Barnard 1961; Brandt et al. 2012; Havermans 2016), and at hadal depths (Thurston et al. 2002; Fujii et al. 2013; Eustace et al. 2016; Weston et al. 2020a, 2021). They have been the focus of ecological and physiological studies, including metabolism (Premke and Graeve 2009), feeding strategies (Hargrave 1985; Premke et al. 2006; Blankenship and Levin 2007), population demographics (Ingram and Hessler 1987; Christiansen et al. 1990; Thurston et al. 2002; Blankenship et al. 2006), and biomonitoring (Reid et al. 2018). However, most studies have presumed to be studying *Eurythenes gryllus* (Lichtenstein in Mandt, 1822). Questions to the identification of *E. gryllus* were first raised by France and Kocher (1996). Cryptic speciation with the *gryllus*-complex has since been confirmed by integrative taxonomic studies (Havermans et al. 2013; Havermans 2016; Eustace et al. 2016). Since 2015, *Eurythenes* has expanded from three to nine described species (d’Udekem d’Acoz and Havermans 2015; Narahara-Nakano et al. 2018; Weston et al. 2020a). Furthermore, at least five distinct genetic lineages are awaiting formal description (France and Kocher 1996; Havermans et al. 2013; Eustace et al. 2016; Horton et al. 2020) and more are likely to be discovered via expansion of sampling programs (Havermans 2016).

**Fig. 1:**
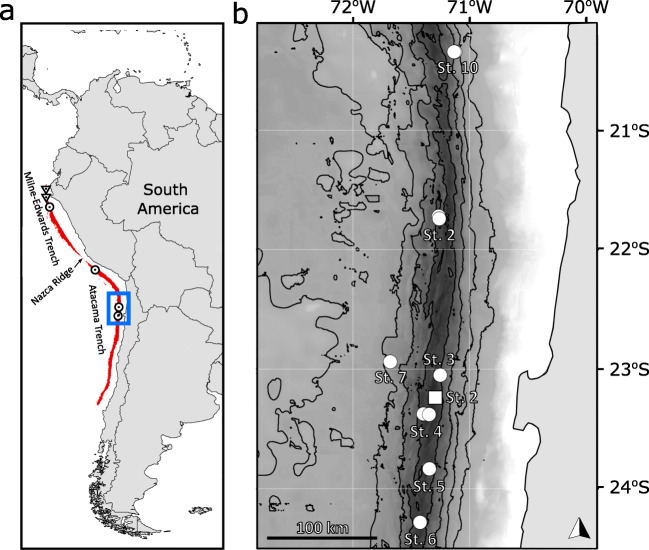
**a** Map of the Peru-Chile Trench defined by depths >4900 m (red). Historical collection records of this species (circle), and the historical abyssal sampling with the absence of *Eurythenes atacamensis* sp. nov. (triangle). The extent of map (**b**) is indicated by the blue box. **b** The eleven deployments where *E. atacamensis* sp. nov. was recovered in the Atacama Trench during the Atacamex Expedition (square) and the RV Sonne SO216 Expedition (circle). Isobaths are shown every 1000 m between 3000- and 7000-m-depth contours.

One undescribed lineage is from hadal depths in the Peru-Chile Trench, eastern South Pacific Ocean (Thurston et al. 2002; Ritchie et al. 2015; Eustace et al. 2016). This species was first recorded from 7196 m by *in situ* still images during the Scripps Institution of Oceanography Expedition SOUTHTOW (Hessler et al. 1978). The first specimens were recovered via baited traps from 7230 m during SIO BI72–20 (Ingram and Hessler 1987) and subsequently from 7800 m in September 1997 during the Atacama Trench International Expedition (ATIE; Thurston et al. 2002). In these three studies, specimens were identified as *E. gryllus*. However, distinct morphological differences from the *E. gryllus* description were observed with the gnathopods, coxa 4, and epimeron 3 (Thurston et al. 2002). These differences were proposed to indicate the population was undergoing incipient speciation. Based on specimens from the 2010 RV Sonne SO209 expedition, a combined morphological and molecular identification approach resolved that this population is a distinct lineage, *Eurythenes* sp. ‘PCT hadal’ (Ritchie et al. 2015; Eustace et al. 2016). This undescribed species is considered to be restricted to hadal depths (6173–8074 m) of the Peru-Chile Trench (Eustace et al. 2016), which is partitioned by the ~4000-m-deep Nazca Ridge to northern (Milne-Edwards Trench) and southern (Atacama Trench) sectors (Fig. 1; Hampel et al. 2004). The pattern of ontogenetic vertical stratification across the depth gradient was found, whereby juveniles were prevalent at shallower depths and females dominated the deepest depths (Eustace et al. 2016). However, the SO209 Expedition specimens were recovered from only three sampling locations widely spaced along the north-south axis of the trench.

This present study is based on specimens collected using baited landers across abyssal to hadal depths of the Atacama Trench during the 2018 RV Sonne SO261 Expedition and at the deepest point as part of the 2018 Atacamex Expedition. We applied an integrative taxonomic approach to describe the tenth species of *Eurythenes*, namely, *Eurythenes atacamensis* sp. nov., and provided an updated key for the genus. Further, we investigated morphometric and bathymetric trends related to size and ontogeny across a latitudinally focused sampling transect.

## Material and methods

### Specimen collection and processing

Specimens were collected during two expeditions in 2018 focused on the abyssal and hadal depths of the Atacama Trench off northern Chile (24–21°S). The Atacamex Expedition was during January–February 2018 onboard the RV Cabo de Hornos, and the RV Sonne SO261 Expedition was conducted during March 2018 as part of the HADES-ERC project (Wenzhöfer 2019). Both expeditions deployed baited free-fall landers. The Atacamex Expedition used a custom-design Nano Lander from Global Ocean Design (San Diego, CA) named ‘Audacia’—equipped with a baited mesh catching trap, a conductivity-temperature-depth-oxygen (CTD-O) profiler, a small video camera, and two 30-L Niskin bottles. The ‘Audacia’ was recovered after 24 h using an acoustic releaser. The RV Sonne SO261 Expedition deployed two landers, Camera Lander 1 and Lander 2, between depths of 2548 and 8052 m. The Camera Lander 1 and 2 were equipped with an RBRduet3 TD pressure sensor (RBR, Canada) and a bespoke funnel trap. The trap was an acrylic tube (20 cm diameter and 100 cm long) with a funnel (5 cm diameter) at one end and a 1-mm steel mesh at the other end. When the ballast weight was released, the funnel was plugged to minimize the loss of samples during surfacing and recovery. The traps were baited with whole-bait mackerel (Scombridae; Jamieson et al. 2011). Pressure records were converted to depth (m) following Saunders (1981). The seven abyssal and hadal stations from the RV Sonne SO261 Expedition and a single station from the Atacamex Expedition are shown in Fig. 1, and the details for the entire 11 deployments are provided in Table 1.

**Table 1:** Collection information for *Eurythenes atacamensis* sp. nov. during the Atacamex and RV Sonne SO261 Expeditions

Depth (m)	Latitude	Longitude	Expedition	Station	Date	Female	Male	Intersex	Juvenile
4974	22° 56.282′ S	71° 40.686′ W	SO261	7	20/03/2018	–	–	–	15
5920	20° 20.608′ S	71° 07.821′ W	SO261	10	27/03/2018	–	–	–	16
6025	20° 20.610’ S	71° 07.824′ W	SO261	10	27/03/2018	–	–	–	20
6520	21° 43.200′ S	71° 15.813′ W	SO261	2	24/03/2018	3	–	–	56
6714	21° 44.497′ S	71° 15.465′ W	SO261	2	24/03/2018	14	–	–	103
7139	23° 02.998′ S	71° 15.044′ W	SO261	3	18/03/2018	5	–	–	1
7204	23° 22.384′ S	71° 23.577′ W	SO261	4	14/03/2018	117	1	–	88
7493	23° 49.981′ S	71° 20.635′ W	SO261	5	12/03/2018	20	–	–	9
7834	24° 16.504′ S	71° 25.388′ W	SO261	6	08/03/2018	60	–	1	3
8052	23° 22,774′ S	71° 20.683′ W	SO261	4	14/03/2018	138	–	–	8
8081	23° 24.48′ S	71° 19.91′ W	Atacamex	2	30/01/2018	2	–	–	–
					Total	356	1	1	319

Included is the number of individuals collected by sex for each depth

On the Atacamex Expedition, amphipods were preserved with 95% ethanol. The ethanol was replaced within 24 h of initial preservation, and the samples were subsequently stored at −20 °C. On the RV Sonne SO261 Expedition, amphipods were preserved in 70% ethanol upon initial sorting on deck. Whole-type specimens were photographed with a Canon EOS 750D DSLR camera, Tamron SP 90mm f/2.8 VC USD Macro 1:1 VC Lens with a polarising filter, and Falcon Eyes CS-730 copy stand and processed with Helicon Focus and Helicon Remote software (Helicon Soft). Appendages were dissected using a stereomicroscope (Wild Heerbrugg M8), temporarily mounted with glycerol, and imaged with a Leica DMi8 inverted microscope and DFC295 camera. The length of appendage articles was measured from the proximal to the distal articular condyle (or closest estimated position) following Horton and Thurston (2014) to control for the degree of limb flexing. Images were digitally inked following a method adapted from Coleman (2003, 2009) using Inkscape v0.92.2.

The type material was selected post-expedition. The holotype was selected from among the DNA barcoded specimens as to minimize the potential for future taxonomic and nomenclature issues (d’Udekem d’Acoz and Havermans 2015; Weston et al. 2020b). Type material is deposited at the Museo Nacional de Historia Natural, Santiago, Chile (MNHNCL) and the Zoological Museum, Universidad de Concepción, Chile (MZUC). GenSeq nomenclature is applied to type material following Chakrabarty et al. (2013).

### DNA barcoding and phylogenetics

The phylogenetic placement of *E. atacamensis* sp. nov. within the genus was assessed at two partial mitochondrial barcoding regions, 16S rRNA (16S; 260 bp) and cytochrome c oxidase subunit I (COI; 624 bp), for specimens collected on both expeditions. For the Atacamex Expedition, total genomic DNA was extracted from a single specimen using the Forensic DNA Kit (Omega) based on the manufacturer protocol, except for incubation in the lysis buffer and proteinase K overnight. For the RV Sonne SO216 specimens, the Bioline ISOLATE II Genomic DNA Kit was used to extract total genomic DNA from the pleopods of five *E. atacamensis* sp. nov. specimens collected between 4974 and 8052 m (Table 2). DNA was extracted from comparative specimens of *Eurythenes maldoror* d’Udekem d’Acoz & Havermans, 2015 and one of *Eurythenes magellanicus* (H. Milne Edwards, 1848), both recovered from 4974 m at station 7 (Table 2). The primer sets used for amplification were AMPH1 (France and Kocher 1996) and ‘Drosophila-type’ 16SBr (Palumbi et al. 2002) for 16S and LCO1490 and HCO12198 (Folmer et al. 1994) for COI. PCR protocols were followed as described in Ritchie et al. (2015). Sequences were cleaned enzymatically using New England Biolabs Exonuclease 1 and Antarctic Phosphatase.

**Table 2:** Species, GenBank sequence accession numbers, and references for phylogenetic analysis of *Eurythenes atacamensis* sp. nov..

Species	16S	COI	Reference
*Alicella gigantea*	KP456083	KP713893	Ritchie et al. 2015
*Eurythenes aequilatus*	LC229090	LC229094	Narahara-Nakano et al. 2018
*Eurythenes aequilatus*	LC229091	LC229095	Narahara-Nakano et al. 2018
*Eurythenes andhakarae*	JX887065	JX887114	Havermans et al. 2013
*Eurythenes andhakarae*	JX887066	JX887119	Havermans et al. 2013
*Eurythenes atacamensis* sp. nov.	MW042880	No amp	This study (4974 m; genseq-2)
*Eurythenes atacamensis* sp. nov.	MW042881	MW048993	This study (5920 m; genseq-2)
*Eurythenes atacamensis* sp. nov.	MW042882	MW048994	This study (7139 m; genseq-2)
*Eurythenes atacamensis* sp. nov.	MW042883	No amp	This study (7834 m; genseq-2)
*Eurythenes atacamensis* sp. nov.	MW042884	MW048996	This study (8052 m; genseq-1)
*Eurythenes atacamensis* sp. nov.	MW290039	MW288146	This study (8081 m; genseq-2)
*Eurythenes gryllus*	JX887060	JX887132	Havermans et al. 2013
*Eurythenes gryllus*	JX887063	JX887136	Havermans et al. 2013
*Eurythenes magellanicus*	LC192879	LC192881	Narahara-Nakano et al. 2018
*Eurythenes magellanicus* (‘Eg5’)	JX887071	JX887144	Havermans et al. 2013
*Eurythenes magellanicus*	JX887074	JX887145	Havermans et al. 2013
*Eurythenes magellanicus*	No data	KX078274	Havermans 2016
*Eurythenes magellanicus*	MW042879	No amp	This study (4974 m)
*Eurythenes maldoror*	JX887069	JX887151	Havermans et al. 2013
*Eurythenes maldoror*	JX887068	JX887152	Havermans et al. 2013
*Eurythenes maldoror*	JX887067	JX887121	Havermans et al. 2013
*Eurythenes maldoror*	KX034310	KX365240	Ritchie et al. 2017
*Eurythenes maldoror*	MW042878	MW048992	This study (4974 m)
*Eurythenes obesus*	KP456144	KP713954	Ritchie et al. 2015
*Eurythenes obesus*	No data	Eob-C103	d’Udekem d’Acoz and Havermans 2015
*Eurythenes plasticus*	MT021437	MT038070	Weston et al. 2020a
*Eurythenes plasticus*	MT021438	MT038071	Weston et al. 2020a
*Eurythenes plasticus*	MT021439	MT038072	Weston et al. 2020a
*Eurythenes plasticus* (‘Eg7’)	U40445	No data	France and Kocher 1996
*Eurythenes sigmiferus*	JX887070	No data	Havermans et al. 2013
*Eurythenes sigmiferus*	AY943568	No data	Escobar-Briones et al. 2010
*Eurythenes thurstoni*	U40449	No data	France and Kocher 1996
*Eurythenes* sp. ‘Eg8’	U40439	No data	France and Kocher 1996
*Eurythenes* sp. ‘Eg8’	U40440	No data	France and Kocher 1996
*Eurythenes* sp. ‘Eg9’	U40446	no data	France and Kocher 1996
*Eurythenes* sp. ‘Eg9’	U40448	No data	France and Kocher 1996
*Eurythenes* sp. ‘PAP’	No data	MN832603	Horton et al. 2020
*Eurythenes* sp. ‘PAP’	No data	MN832604	Horton et al. 2020
*Eurythenes* sp. ‘PCT abyssal’	KP456140	KP713957	Ritchie et al. 2015
*Eurythenes* sp. ‘PCT abyssal’	KP456141	KP713958	Ritchie et al. 2015
*Eurythenes* sp. ‘PCT hadal’	KP456138	KP713955	Ritchie et al. 2015 (7050 m)
*Eurythenes* sp. ‘PCT hadal’	KP456139	KP713956	Ritchie et al. 2015 (7050 m)
*Eurythenes* sp. ‘PCT hadal’	KR527251	No data	Eustace et al. 2016
*Eurythenes* sp. ‘PCT hadal’	KR527252	No data	Eustace et al. 2016

No amp. means either no PCR product or sequence. Included for this study are specimen recovery depth (m) and GenSeq ranking

The RV Sonne SO216 PCR products were sequenced with an ABI 3730XL sequencer (Eurofins Genomics, Germany), and the Atacamex Expedition PCR products were sequenced by the sequencing service of P. Universidad Católica de Chile. Electropherograms were confirmed and trimmed by eye in MEGA 7 (Kumar et al. 2016). Nucleotide sequence quality and absence of contamination were verified on NCBI BLASTn. Each COI sequence was translated into their amino acid sequence to assess for stop codon presence.

The phylogenetic relationship of *E. atacamensis* sp. nov. within *Eurythenes* was investigated with publicly available data in two datasets, namely, 16S and COI. The comparative sequences were selected to represent type material, high-confident identifications, or from defined undescribed lineages (Table 2; France and Kocher 1996; Escobar-Briones et al. 2010; Havermans et al. 2013; d’Udekem d’Acoz and Havermans 2015; Ritchie et al. 2015; Havermans 2016; Narahara-Nakano et al. 2018; Ritchie et al. 2017; Horton et al. 2020; Weston et al. 2020a). The sequences associated with *Eurythenes* cf. *thurstoni* (KX078272), *Eurythenes* n. sp. 1 (KX078273), and *Eurythenes* n. sp. 2 (KX078271) from Havermans (2016) were excluded from the COI alignment due to low percent identity (<70%) with other *Eurythenes* in NCBI BLASTn search. *Alicella gigantea* Chevreux, 1899 was selected as the outgroup for both genes in the phylogenetic analysis, as it is a large deep-sea scavenger in a separate superfamily with sufficient phylogenetic distance (Table 2; Lowry and De Broyer 2008; Ritchie et al. 2015). Sequence alignments were constructed by MAFFT v7 using default parameters (Katoh et al. 2017). The final 16S alignment consisted of 41 individuals from nine *Eurythenes* species, four genetic, undescribed lineages, and the outgroup. The final alignment for COI consisted of 31 individuals from seven described species, three genetic, undescribed lineages, and the outgroup.

Phylogenetic relationships were inferred via a Bayesian Inference (BI) using the Bayesian Evolutionary Analysis by Sampling Trees (BEAST) software package v1.10.4 (Suchard et al. 2018) and a maximum likelihood (ML) phylogenetic analysis with PhyML v3.1 (Guindon et al. 2010). The optimal evolutionary models were identified in MEGA 7 based on by the Bayesian information criterion (BIC) as the HKY + G model for 16S and the HKY + I + G model for COI (Hasegawa et al. 1985). On BEAST, two independent runs of 40,000,000 generations were conducted by sampling every 10,000 generations using an uncorrelated relaxed clock (Drummond et al. 2012). Model convergence was assessed in Tracer v1.7 (ESS > 200; Rambaut et al. 2018). The first 10% of states were discarded. The maximum clade credibility tree was generated using TreeAnnotator v1.8.4 (Drummond et al. 2012), viewed in FigTree v1.4.3, and annotated using Inkscape v0.92.2 (https://inkscape.org). The ML analysis was setup with a neighbour-joining starting tree and interchange branch swapping using the model of sequence evolution and parameters estimated by PhyML (Guindon et al. 2010; http://www.atgc-montpellier.fr/phyml/). The node stability was based on bootstrap support with 10,000 iterations.

Two analytical approaches were used for delimiting the *Eurythenes* species, namely, the Generalized Mixed Yule Coalescent (GMYC) likelihood method and the Bayesian Poisson Tree Process (bPTP) model. For the GYMC analysis, the following parameters were selected: the GTR nucleotide substitution model for COI and HKY for 16S, a normalized exponential relaxed clock, and a Yule process of speciation for both genes. Three independent runs were performed to ensure convergence. Each run was conducted for 10^9^ generations, and every 10,000 generations were sampled. The output files were visualized in Tracer v1.4 to determine the convergence of the chains (ESS >200; Rambaut et al. 2018). The maximum clade credibility (MCC) tree was determined by TreeAnnotator BEAST v2.6.2 (Bouckaert et al. 2019), after burning the first 25% of the trees. The number of delimited species was determined using each MCC gene tree through the ‘gymc’ function in the *splits* package in R (Ezard et al. 2017). Model results were evaluated from a likelihood ratio test that calculates significance from the chi-square test. The bPTP model was used to infer species boundaries through the PTP webserver (Zhang et al. 2013; http://species.h-its.org/ptp/). The BI derived 16S and COI topologies were used as the input tree. The bPTP analysis was conducted for 100,000 generations of MCMC sampling, with a thinning value of 100 and burn-in of 25%.

### Morphometric relationships and bathymetric trends

Bathymetric trends were assessed in relation to sex for the RV Sonne SO261 specimens. Males were identified by the presence of penile papillae, and females were identified by the presence of oostegites. Intersex was classified by the presence of both oostegites and penile papillae. Juveniles were classified by the visual absence of oostegites and penile papillae (Ingram and Hessler 1987; Eustace et al. 2016). Total body length (rostrum to the end of telson) and coxa 4 length (diagonal) were measured to the nearest 0.1 mm using digital callipers (Fisher Scientific; Duffy et al. 2016; Lacey et al. 2018). Individuals were weighed to the nearest 0.001 g, following 1 min of drying.

The total length-weight relationship was calculated using all individuals from the RV Sonne SO261 between 6714 and 8052 m. The relationship was based on the following non-linear formula: *W* = *a* × *TL*^*b*^, where *w* is weight, TL is total length, and *a* and *b* are regression-derived parameters. The total length-coxa 4 relationship was examined using an ordinary least squares linear regression, with nearly the same set of individuals as the total length-weight relationship, apart from the intersex individual. The model assumptions were checked for normality and heterogeneity of variance using histograms of the residuals and by examining qqplots and the fitted values versus residuals. The relationship between total length by depth for females was examined using Spearman correlation. The relationship between total length and depth was sigmoidal for juveniles. As such, a non-linear 4-part self-starting logistic regression was fit using the package *nlme* v3.1 (Pinheiro et al. 2020). The analysis was conducted in R version 3.6.3.

## Results

### Systematics

**Order Amphipoda Latreille, **1816

**Superfamily Lysianassoidea Dana,** 1849

**Family Eurytheneidae Stoddart and Lowry,** 2004

**Genus** ***Eurythenes*** **S. I. Smith in Scudder,** 1882

**Fig. 2:**
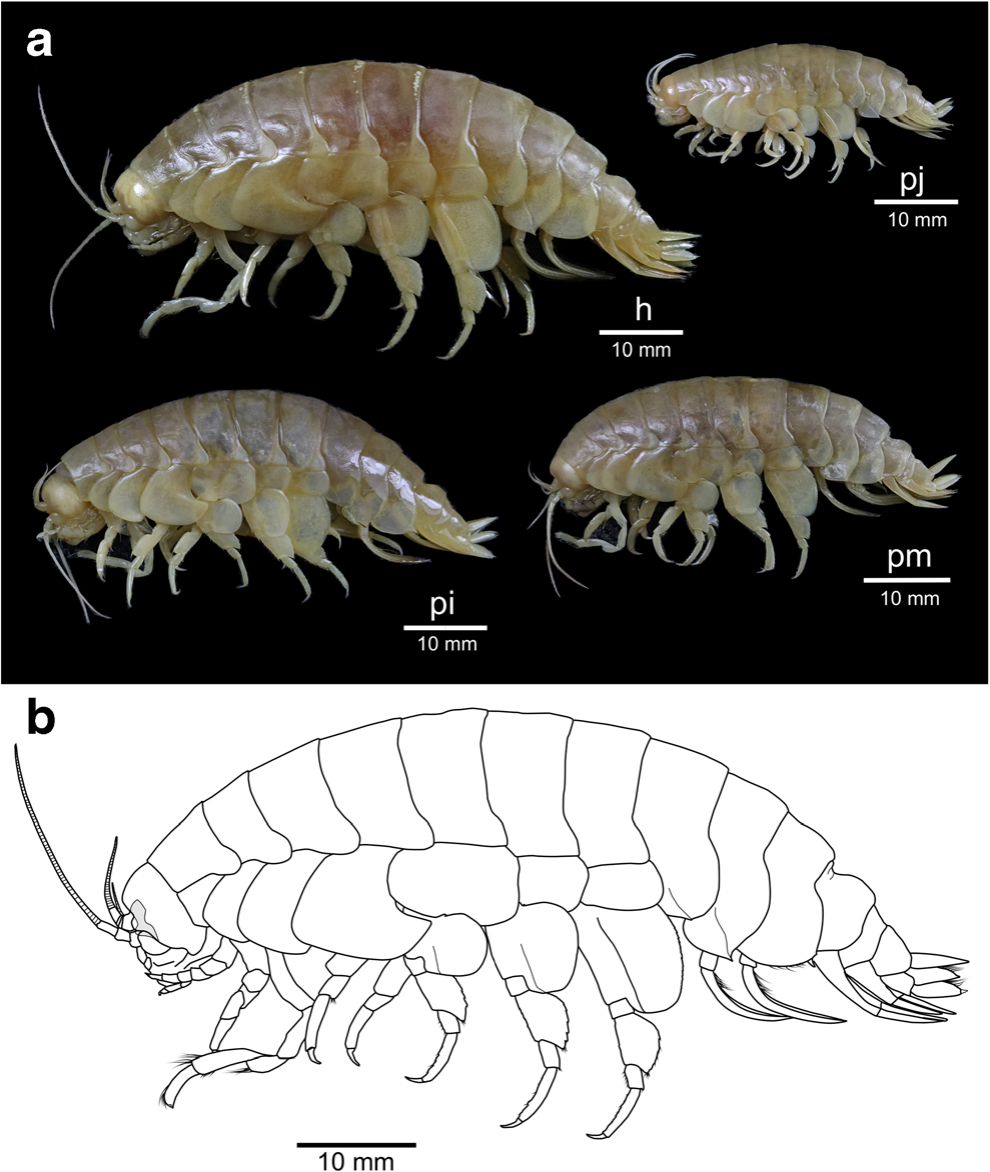
**a** *Eurythenes atacamensis* sp. nov.: female holotype from 8052 m (h; MNHNCL AMP-15816), juvenile paratype from 6714 m (pj; MNHNCL AMP-15818), intersex paratype from 7834m (pi; MNHNCL AMP-15820), male paratype from 7204 m (pm; MNHNCL AMP-15817); **b** *Eurythenes atacamensis* sp. nov., mature female, holotype, MNHNCL AMP-15816

**Fig. 3:**
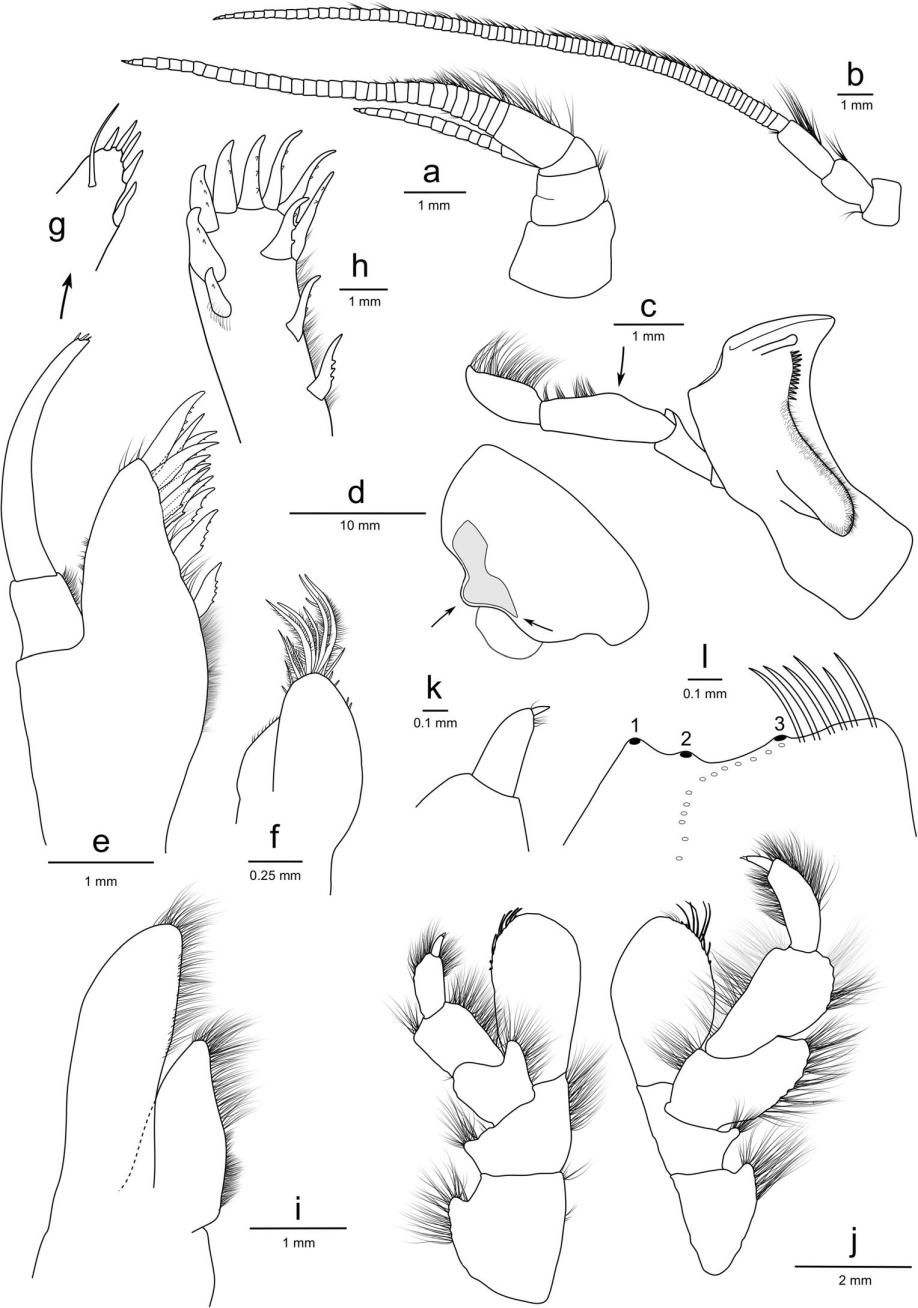
*Eurythenes atacamensis* sp. nov. holotype (MNHNCL AMP-15816). **a** left antenna 1; **b** left antenna 2; **c** left mandible with an arrow to highlight the broad palp; **d** head with arrows to highlight the anterior lobe and ventral corner of the eye; **e** left maxilla 1 outer plate and palp not flattened; **f** left maxilla 1 inner plate; **g** left maxilla 1 palp insert; **h** left maxilla 1 outer plate face; **i** left maxilla 2; **j** left and right maxillipeds with inner plates removed; **k** left maxilliped dactylus insert; **l** left maxilliped inner plate (medio-facial spines not shown)

**Fig. 4:**
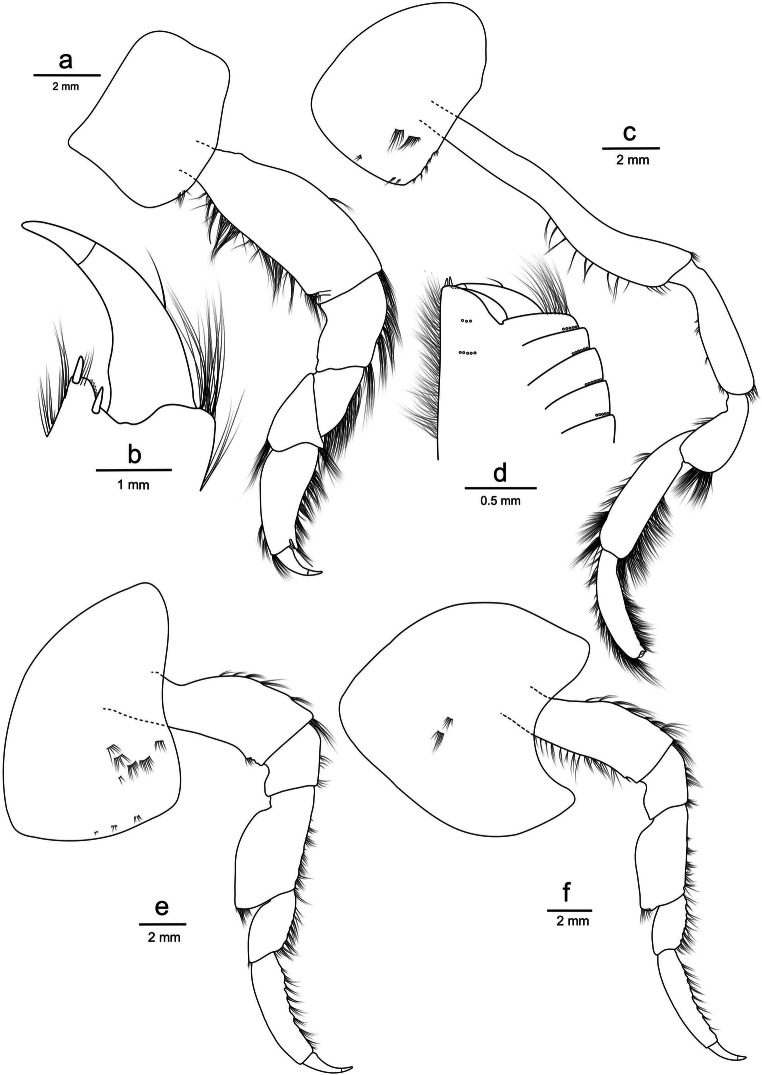
*Eurythenes atacamensis* sp. nov. holotype (MNHNCL AMP-15816). **a** left gnathopod 1; **b** chela of left gnathopod 1; **c** left gnathopod 2; **d** chela of left gnathopod 2; **e** left pereopod 3; **f** left pereopod 4

**Fig. 5:**
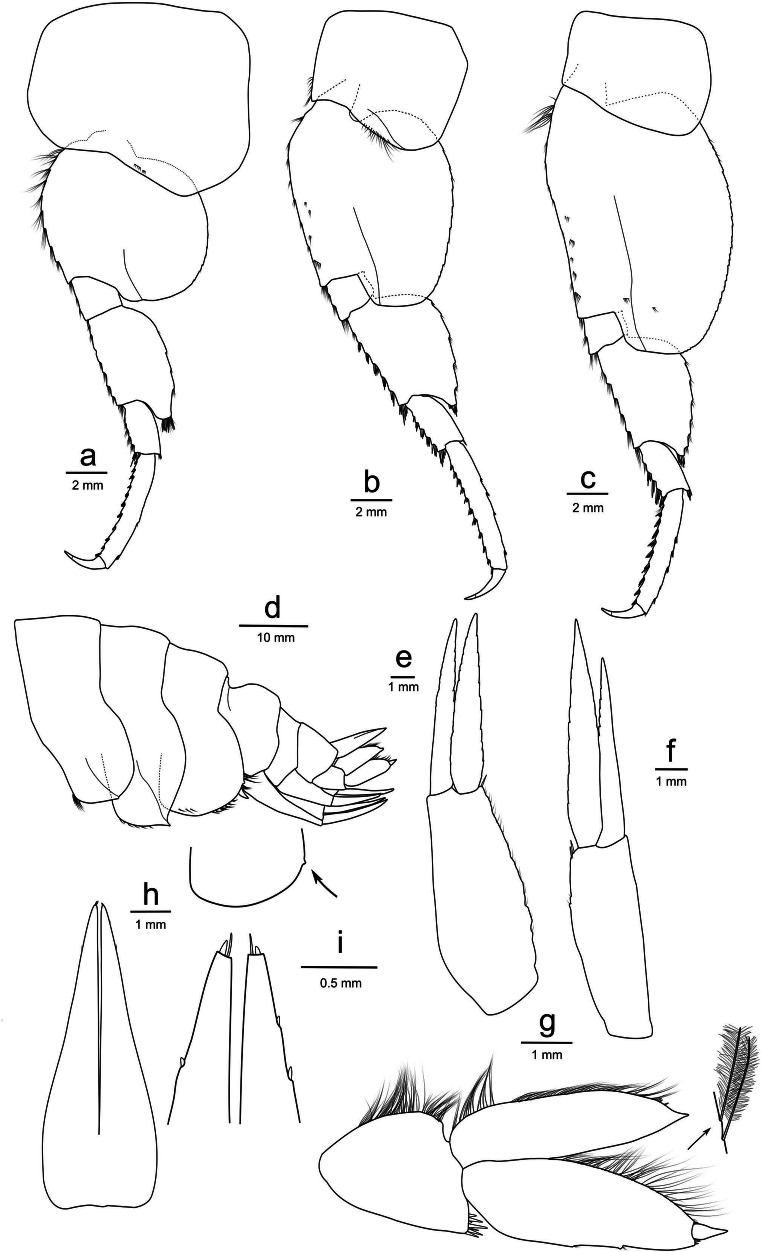
*Eurythenes atacamensis* sp. nov. holotype (MNHNCL AMP-15816). **a** left pereopod 5; **b** left pereopod 6; **c** left pereopod 7; **d** epimeron and epimeron 3 insert with arrow denoting small tooth on the posteroventral corner; **e** left uropod 1; **f** left uropod 2; **g** left uropod 3 with the arrow showing plumose setae; **h** telson; **i** telson distal margin insert

**Fig. 6:**
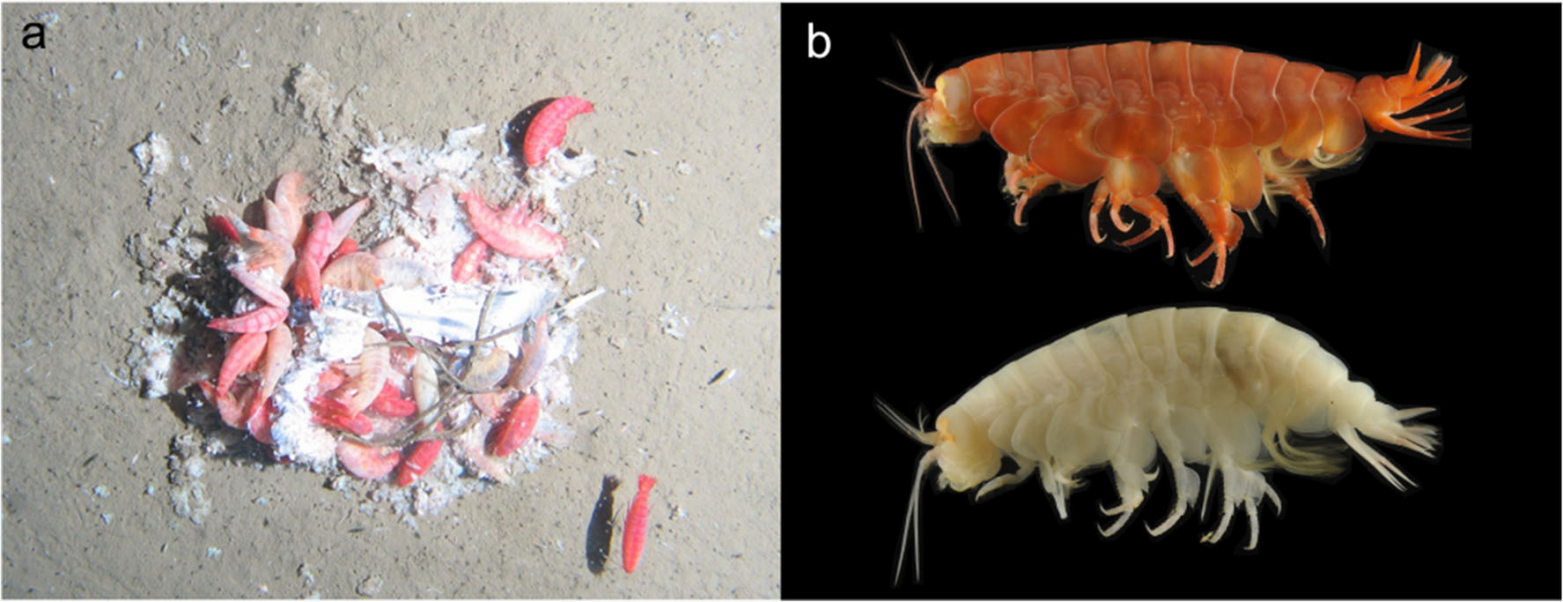
**a** *Eurythenes atacamensis* sp. nov. feeding on bait and **b** two colour morphs prior to ethanol preservation. Still image and specimens are from 8074 m in the Atacama Trench during the 2010 RV Sonne SO209 Expedition (see Eustace et al. (2016) for site location details)

***Eurythenes atacamensis*** **sp. nov. Weston & Espinosa-Leal** (Figures 2–6)

http://zoobank.org/51F715E8-AD60-403C-B39A-06F3A3223935

*Eurythenes gryllus*—Ingram and Hessler 1987: 1889.—Thurston et al. 2002: 205–210, figs. 1–7, table 1.—Jamieson et al. 2019: 1–9, fig. 1, table 1.

*Eurythenes gryllus* Peru-Chile(H)—Ritchie et al. 2015: 121–129, figs.2, 4, tables 1, 2.

*Eurythenes* sp. (Hadal Form)—Eustace et al. 2016: 91–97, fig. 1, fig. 2 (d)(e)(f), fig. 5, tables 2, 3.

**Material Examined.**

**Holotype**: *Female*, total body length 76.2 mm, Atacama Trench, eastern South Pacific Ocean (23° 22.774′ S, 71° 20.683′ W), expedition SO216, station 4, depth 8052 m, MNHNCL AMP-15816, genseq-1 16S (MW042884), COI (MW048996).

**Paratypes**: *Female*, total body length 70 mm, Atacama Trench, Pacific Ocean (23° 24.48′ S, 71° 19.91′ W), Atacamex Expedition, station 2, depth 8081 m, MZUC/UCCC 46674. *Female*, total body length 72 mm, Atacama Trench, Pacific Ocean (23° 24.48′ S, 71° 19.91′ W), Atacamex Expedition, station 2, depth 8081 m, MZUC/UCCC 46675, genseq-2 16S (MW290039), COI (MW288146). *Male*, total body length 50.8 mm, Atacama Trench, Pacific Ocean (23° 22.384′ S, 71° 23.577′ W), expedition SO216, station 4, depth 7204 m, MNHNCL AMP-15817. *Female*, type locality, MNHNCL AMP-15822. *Intersex*, total body length 58.8 mm, Atacama Trench, Pacific Ocean (24° 16.233′ S, 71° 25.386′ W), expedition SO216, station 6, depth 7834 m, MNHNCL AMP-15820, genseq-2 16S (MW042883). *Juvenile*, total body length 16.1 mm, Atacama Trench, Pacific Ocean (21° 44.497′ S, 71° 15.465′ W), expedition SO216, station 2, depth 6738 m, MNHNCL AMP-15819. *Juvenile*, total body length 38.4 mm, Atacama Trench, Pacific Ocean (21° 44.497′ S, 71° 15.465′ W), expedition SO216, station 2, depth 6714 m, MNHNCL AMP-15818. *Juvenile*, Atacama Trench, Pacific Ocean (22° 56.282′ S, 71° 40.686′ W), expedition SO216, station 7, depth 4974 m, MNHNCL AMP-15821.

**Paragenetype**: *Juvenile*, Atacama Trench, Pacific Ocean (22° 56.282′ S, 71° 40.686′ W), expedition SO216, station 7, depth 4974 m, genseq-2 16S (MW042880). *Juvenile*, Atacama Trench, Pacific Ocean (20° 20.608′ S, 71° 07.821′ W), expedition SO216, station 10, depth 5920 m, genseq-2 16S (MW042881), COI (MW048993). *Female*, Atacama Trench, Pacific Ocean (23° 02.998′ S, 71° 15.044′ W), expedition SO216, station 3, depth 7139 m, genseq-2 16S (MW042882), COI (MW048994).

**Type Locality.** Atacama Trench, eastern South Pacific Ocean (23° 22.774′ S, 71° 20.683′ W), expedition SO216, station 4, depth 8052 m.

**Etymology.** The species name, *atacamensis*, references the type locality, Atacama Trench, of this conspicuously abundant scavenging amphipod.

**Diagnosis.** Lateral cephalic lobe rounded and weakly pronounced. Ventral corner of the eye points linearly downwards. Article 2 of mandibular palp expanded posteriorly but not distally tapering. Maxilliped inner plate with three apical, non-protruding nodular setae. Gnathopod 1 subchelate; palm weakly formed, short. Gnathopod 2 minutely chelate; coxa sub-rectangular and posterior margin slightly rounded; palm obtusely angled. Pereopods 3 to 7 dactylus short. Epimeron 3 ventral margin rounded with a small tooth on the posteroventral corner. Uropod 2 inner ramus longer than outer ramus. Lack of dorsal carination or ridging, specifically at pereonite 3.

**Description, based on holotype, female, MNHNCL AMP-15816.**

**Body** (Fig. 2): surface smooth, without setae; urosomite 3 with an anterodorsal depression. *Oostegites* present on gnathopod 2 to pereopod 5, setae absent. *Coxa gills* present on gnathopod 2 to pereopod 7. *Colour pattern* before ethanol preservation unknown as the holotype was selected post-expedition.

**Head** (Fig. 3): rostrum absent; antennal sinus quadrate (Fig. 3d). *Antenna 1* short, 0.13× as long as body length; accessory flagellum 14-articulate; primary flagellum 34-articulate; calceoli absent (Fig. 3a). *Antenna 2* 2.4× the length of antenna 1, 0.25× as long as body; article 4–5 with brush setae; flagellum 68-articulate with some brush setae; calceoli absent (Fig. 3b).

**Mouthpart bundle** (Fig. 3): *Mandible* left lacinia mobilis a long slender robust seta with smooth distal margin; incisor smooth and convex; setal row with 11 short, slender, robust setae; molar large, setose, small triturating surface; palp article-length ratio 1: 1.8: 1.6, article 3 sickle-shaped (Fig. 3c). *Maxilla 1* inner plate with nine apical plumose setae; outer plate with an 8/3-crown arrangement; palp longer than the outer plate, 2-articulate, four apical and one apicolateral robust setae, with one subapical long setae (Fig. 3e–h). *Maxilla 2* both plates broad, inner plate 0.6 × shorter than the outer plate (Fig. 3i). *Maxilliped* inner plate sub-rectangular, three apical, non-protruding nodular setae; outer plate subovate; palp 4-articulate, left and right are asymmetric with right palp exceeding past the outer plate, dactylus well-developed, unguis present (Figure 3j–l).

**Pereon** (Figs. 4 and 5): *Gnathopod 1* coxa sub-quadrate, weakly concave on anterior and ventral margins; basis, long, length 2.2× breadth; palm weakly formed and short (0.1× as long as the posterior margin of propodus), crenulate with one robust seta at base of the palm and another at the end of palm (Fig. 4a–b). *Gnathopod 2* coxa with setae along the posteroventral corner; basis elongate, length 6.9 times width, setae along posterior and ventral margins; posterior margin of merus expanded; propodus sub-rectangular, length 4.5 times width; palm with 2 robust setae on the posterodistal corner; dactylus not reaching palmar corner (Fig. 4c–d). *Pereopod 3* coxa sub-quadrate, 1.5× as long as wide, setae on the surface of coxa and along ventral margin; basis expanded posteriorly, 2.3× as long as wide; merus expanded anteriorly, tuft of setae on the anteroventral corner; carpus stout, 0.6× as long as propodus; propodus 3.9× as long as wide; dactylus slender, short 0.3× as long as propodus, unguis present (Fig. 4e). *Pereopod 4* coxa broad, 0.9× as long as wide, 1.1× length of coxa 3, the junction between anterior and ventral border bluntly angular (sub-rectangular), ventral border straight, posteroventral border weakly oblique; leg almost identical to pereopod 3 (Fig. 4f). *Pereopod 5* coxa sub-rectangular, rounded on both the anterior and posterior margins; basis expanded posteriorly, posterior margin weakly crenulated; merus broadly expanded posteriorly, 1.5× as long as wide, posteroventral margin producing a point; carpus stout, 0.4× as long as propodus; propodus long and slender, 5.5× as long as wide, 11 groups robust setae along anterior margin; dactylus short, 0.4× as long as propodus, unguis present (Fig. 5a). *Pereopod 6* coxa sub-rectangular, setae along the ventral margin, posterior margin straight; basis expanded posteriorly with posterior margin crenulated; merus expanded posteriorly, 1.5× as long as wide, convex posterior margin; propodus and dactylus nearly identical to pereopod 5 (Fig. 5b). *Pereopod 7* coxa sub-rectangular; basis expanded posteriorly, posterior margin distinctly crenulated, distal lobe weakly protruding; merus broad and strongly expanded posteriorly, subequal length to width; propodus and dactylus nearly identical to pereopod 5 (Fig. 5c).

**Pleon and urosome** (Fig. 5): *Epimeron 1* with setae along the anteroventral corner (Fig. 5d). *Epimeron 2* with setae along the ventral margin, posteroventral corner produced into a strong tooth (Fig. 5d). *Epimeron 3* ventral margin rounded with a small tooth on the posteroventral corner (Fig. 5d). *Uropod 1* peduncle with 1 apicomedial seta, rami subequal, outer ramus 0.8× as long as peduncle (Fig. 5e). *Uropod 2* peduncle with 2 apicomedial setae, outer ramus subequal in length to peduncle, inner ramus longer than outer ramus (1.2×; Fig. 5f). *Uropod 3* setae of the distolateral angle of peduncle of normal length and stoutness; inner ramus subequal in length to article 1 of the outer ramus; outer rami article 2 0.8× the length of article 1, medial margins of both rami with plumose setae (Fig. 5g). *Telson* 77% cleft, distal margin of each lobe with one robust and one slender setae (Fig. 5h–i).

#### **Variations**.

Prior to ethanol preservation, body colour of specimens ranged from white, pink, crimson, to dark red and the eye shape and colour were more defined (Fig. 6). This wide variation in body pigmentation is likely attributed to the moult/intermoult cycle (Baldwin and Smith 1987). Minor differences were observed between females and the male. The mature male paratype (MNHNCL AMP-15817) had calceoli present on both antennas 1 and 2. The primary flagellum of antenna 1 was 31-articulate with calceoli present between articles 8 and 20, and the accessory flagellum was 12-articulate. Antenna 2 was 65-articulate. The intersex paratype (MNHNCL AMP-15820) had protruding penile papillae that flexed towards each other but lacked calceoli on antenna 1 or 2. As with the holotype, the oostegites were present on pereopod 2–5; however, the flattened oostegites were not of full length relative to the total body length and lacked setae. Moderate differences were present between sexed and juvenile specimens, with fewer setae on pereopods and uropods and a reduction in articulation on antennae. Specifically, in the juvenile paratype (MNHNCL AMP-15818), the antenna 1 accessory flagellum was 10-articulate, antenna 1 was 26-articulate, and antenna 2 was 57-articulate. Further, the juvenile had more pronounced crenulation of the posterior margin of the basis on pereopods 5–7.

#### **Feeding and distribution**.

This species is a benthopelagic scavenger, which is well documented by its rapid aggregation and feeding at baited the camera landers (Fig. 6a; Hessler et al. 1978). As with *Eurythenes plasticus*, individuals of *E. atacamensis* sp. nov. have been previously documented to ingest microplastics (Jamieson et al. 2019; Weston et al. 2020a). *Eurythenes atacamensis* sp. nov. has a wide bathymetric range (>3000 m) across abyssal to hadal depths (4974–8081 m), including the deepest point of the Atacama Trench. This species is considered to have a distribution localized to both sectors of the Peru-Chile Trench. *Eurythenes atacamensis* sp. nov. is a prominent member of a wider scavenging amphipod community (Fujii et al. 2013). This community is comprised of three species also endemic to the Peru-Chile Trench, *Hirondellea thurstoni* Kilgallen, 2015, *Hirondellea sonne* Kilgallen, 2015, and *Hirondellea wagneri* Kilgallen, 2015.

#### **Differential diagnosis**.

In a genus with cryptic speciation (Havermans et al. 2013), *Eurythenes atacamensis* sp. nov. has distinct diagnostic features. These features include a smooth dorsal body, the palm of gnathopod 1 being very short, and the palm of gnathopod 2 being minutely chelate with an obtusely angled palm. *Eurythenes atacamensis* sp. nov. is the most similar morphologically to *Eurythenes thurstoni* Stoddart & Lowry, 2004, as they both have a minutely chelate gnathopod 2. Yet, *E. atacamensis* sp. nov. can be readily differentiated by the lack of an upturned ridge on the anterodorsal margin of head (present in *E. thurstoni*), uropod 2 inner ramus longer than outer ramus (opposed to subequal), and small tooth on the posteroventral corner of epimeron 3 (versus subquadrate). *Eurythenes thurstoni* is also smaller in total body size, most commonly not longer than 35 mm (Stoddart and Lowry 2004). Additionally, the two species have a disjunct vertical distribution, where *E. thurstoni* lives at bathyal depths (Stoddart and Lowry 2004; d’Udekem d’Acoz and Havermans 2015).

#### Key to *Eurythenes* specimens larger than 25 mm.

This key is expanded from d’Udekem d’Acoz and Havermans (2015), and the caution of use remains. Character differences can be tough to objectively discern, and certain characteristics can be phenotypically variable between cohorts. Visual identification paired with DNA barcoding is strongly recommended.

1. Dactylus of pereopods 3–7 short (less than 0.3 of propodus)…2
  Dactylus of pereopods 3–7 long (more than 0.6 of propodus)…*Eurythenes obesus* (Chevreux, 1905)
2. The palm of gnathopod 2 minutely chelate or very protruding…3
  The palm of gnathopod 2 subchelate or weakly protruding…4
3. The anterodorsal margin of the head forming an upturned ridge; posterodistal lobe of the basis of pereopod 7 very long…*Eurythenes thurstoni* Stoddart & Lowry, 2004
  The anterodorsal margin of the head not forming an upturned ridge; palm of gnathopod 1 very short; posterodistal lobe of the basis of pereopod 7 short or fairly short…*Eurythenes atacamensis* sp. nov.
4. Pereopods 6–7 and epimerons 1–3 not dorsally keeled to slightly keeled; pereopods 6–7 and epimerons 1–2 dorsally not sigmoid (without anterior concavity), epimeron 3 with distinct anterior concavity…5
  Pereopods 6–7 and epimerons 1–3 dorsally strongly keeled and sigmoid (anteriorly slightly to distinctly concave)…*Eurythenes sigmiferus* d’Udekem d’Acoz & Havermans, 2015
5. Eyes of variable width; the outer plate of maxilla 1 with 8/3 crown arrangement…6
  Eyes of constant width; the outer plate of maxilla 1 with 9/3 crown arrangement…*Eurythenes aequilatus* Narahara-Nakano, Nakano & Tomikawa, 2017
6. Article 2 of mandibular palp moderately to strongly expanded posteriorly…7
  Article 2 of mandibular palp not to weakly expanded posteriorly…8
7. Maxilliped with 3 non-protruding nodular spines; pereopod 7 with basis posteriorly strongly expanded, with merus narrow…*Eurythenes andhakarae* d’Udekem d’Acoz & Havermans, 2015
  Maxilliped with 8–9 non-protruding nodular spines; pereopod 7 with basis posterior border weakly expanded, with merus stout…*Eurythenes maldoror* d’Udekem d’Acoz & Havermans, 2015
8. Gnathopod 2 palm convex; uropod 1 and 2 rami subequal…9
  Gnathopod 2 palm straight; the outer ramus of uropod 1 and 2 are shorter than paired inner ramus… *Eurythenes magellanicus* (H. Milne Edwards, 1848)
9. Ventral corner of eye rounded and obliquely pointing backward; maxilliped with 3–4 protruding nodular spines; gnathopod 1 palm convex…*Eurythenes plasticus* Weston, 2020
  Ventral corner of eye sharp and pointing downward; maxilliped with 3–4 non-protruding nodular spines; gnathopod 1 palm straight…*Eurythenes gryllus* (Lichtenstein in Mandt, 1822)

### DNA barcoding and phylogenetics

Thirteen sequences have been annotated, deposited on GenBank, and assigned GenSeq nomenclature (Table 2; 16S: MW042878–84, MW290039 and COI: MW048992–96, MW288146; ncbi.nlm.nih.gov/genbank). Four *E. atacamensis* sp. nov. and one *E. maldoror* were successfully characterized across both 16S and COI (Table 2). Two *E. atacamensis* sp. nov. and one *E. magellanicus* were only characterized across 16S (Table 2). The depths of the *E. atacamensis* sp. nov. specimens spanned from 4974 to 8081 m.

**Fig. 7:**
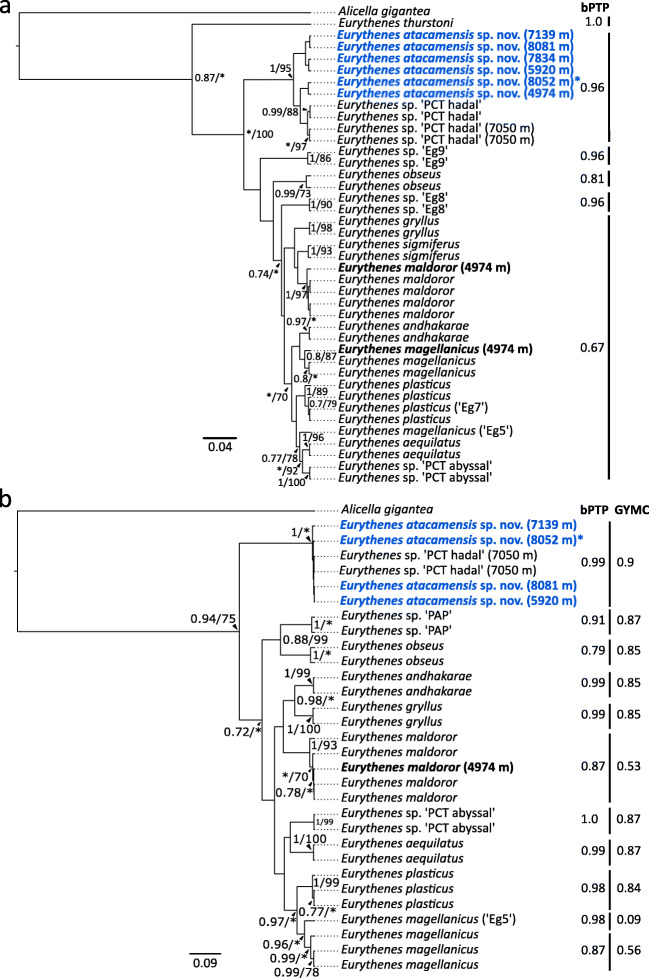
Bayesian phylogenies showing the relationship of *Eurythenes atacamensis* sp. nov. within *Eurythenes* based on **a** 16S rRNA and **b** COI. Specimens added by this study are in bold, with *E. atacamensis* sp. nov. in blue. An asterisk next to the name denotes holotype. References for comparative sequences are in Table 2. Branch nodes have Bayesian posterior probabilities and maximum likelihood bootstrap support values. Values less than 0.7 or 70 are not stated or depicted by an asterisk. Species delimitation inferences by the bPTP and/or GYMC analyses are shown on the right side of each phylogeny.

The phylogenetic relationship of *E. atacamensis* sp. nov. within *Eurythenes* was studied separately for 16S and COI genes (Fig. 7). The *E. atacamensis* sp. nov. specimens of this study were placed within the same undescribed clade as those presented in Ritchie et al. (2015) and Eustace et al. (2016), namely, the *Eurythenes* sp. ‘PCT hadal’, with high support values (16S: BI =0.62, ML = 100; COI: BI = 0.94, ML = 75). This clade was repeatedly placed more basal in the phylogenies. In the 16S topology, only *E. thurstoni* was basal to *E. atacamensis* sp. nov. The *E. atacamensis* sp. nov. clade in the 16S topology had two subclades; however, this distinction was not present in the COI phylogeny. Within the *E. atacamensis* sp. nov. clade, there was a lack of apparent patterns based on depth or station proximity to the trench axis.

The species delimitation analysis showed agreement among the individual phylogenies to support multiple species being present within *Eurythenes*. For 16S, the bPTP analysis inferred six species (mean, 6.78; acceptance rate, 0.179; the estimated number of species, 5–16). However, no distinct entities were differentiated by the GYMC analysis (*p*>0.436), due to low support values (<0.7). There was bPTP support for *E. atacamensis* sp. nov. to be a discrete lineage (0.96; Fig. 7a). The bPTP model estimated 11 species of *Eurythenes* within the COI topology (mean, 12.58; acceptance rate, 0.13; the estimated number of species, 10–16). In concordance, GMYC found 11 distinct entities to be associated with the highest likelihood score (confidence interval 11–18; *p* < 0.005). *Eurythenes atacamensis* sp. nov. was delineated into a distinct lineage by both analyses (bPTP: 0.99; GMYC: 0.9; Fig. 7b).

### Morphometric relationships and bathymetric trends

**Fig. 8:**
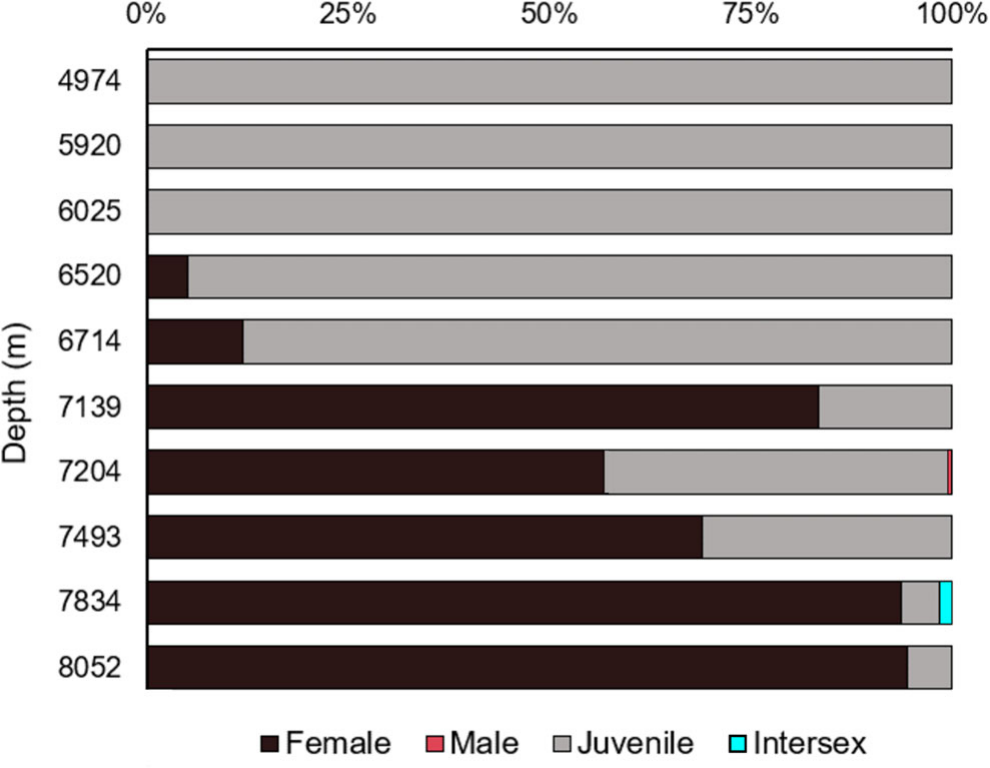
The relative proportion of females, males, juveniles, and intersex of *Eurythenes atacamensis* sp. nov. by depth (m) at the Atacama Trench

A total of 677 specimens of *E. atacamensis* sp. nov. were recovered from the 11 stations (Table 1). None of the 319 females had setae on oostegites or were found to be ovigerous. A single male and a single intersex individual were recovered from 7204 to 7834 m, respectively (Fig. 8). The 356 juveniles were found across the entire depth range sampled (4974–8052 m) and dominated in relative abundance at 6714 m and shallower (88–100%; Fig. 8). In contrast, females were found between 6520 and 8052 m and increased in relative abundance with depth from 5 to 95% (Fig. 8).

**Fig. 9:**
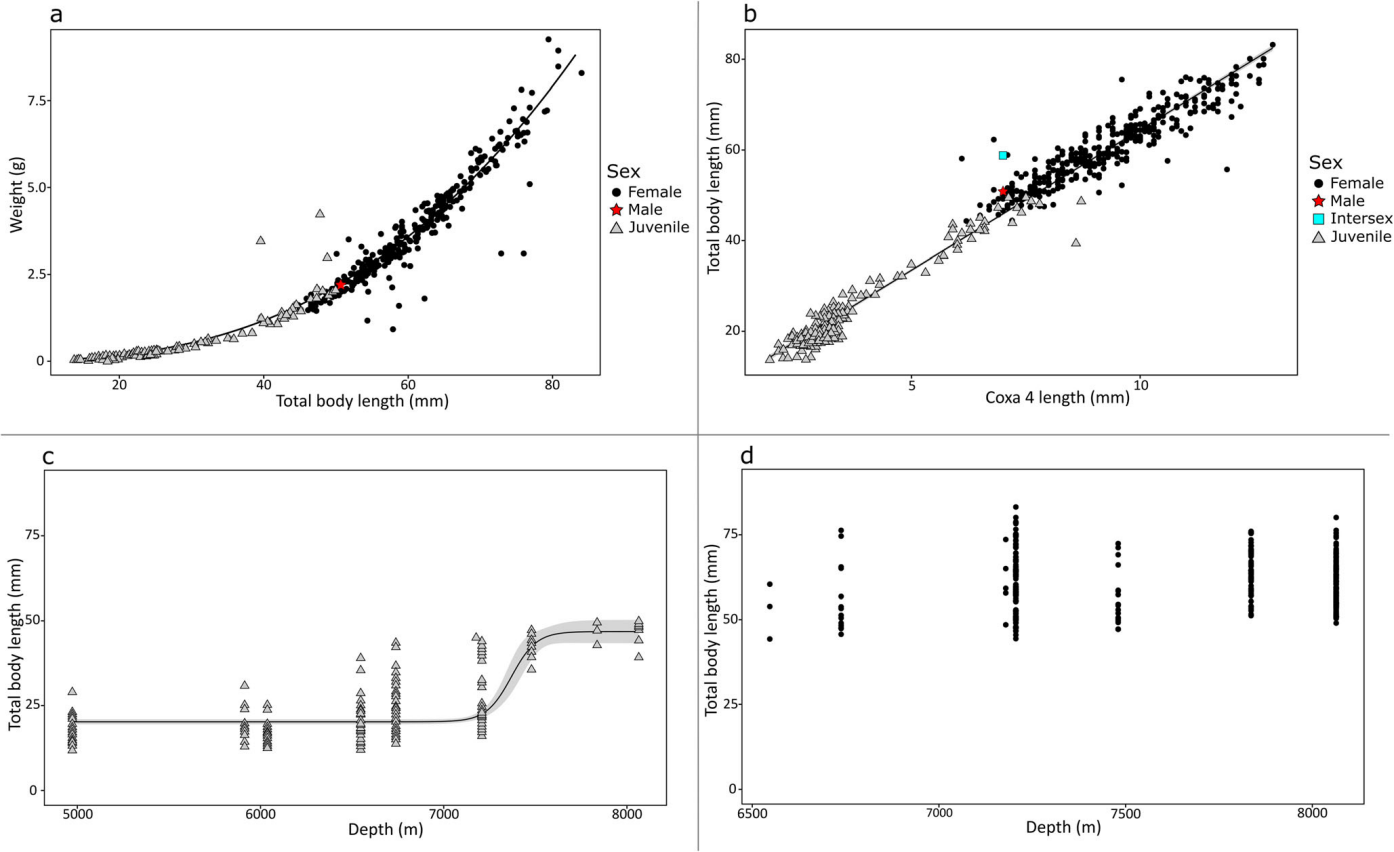
Morphometric relationship between **a** total body length and weight and **b** coxa 4 diagonal length and total body length. Bathymetric relationship of total body length for **c** juvenile and **d** female *Eurythenes atacamensis* sp. nov. Grey areas in **b** and **c** represent 95% confidence intervals of the model mean

Female *E. atacamensis* sp. nov. ranged in total length from 44.3 to 83.2 mm and weight from 1.09 to 9.10 g (Fig. 9a, b). Juveniles ranged in total length from 12.1 to 49.9 mm and ranged in weight from 0.042 to 4.22 g (Fig. 9a, b). The only male specimen measured 50.8 mm and weighed 2.18 g (Fig. 9a, b), and the only intersex individual measured 58.8 mm in total length, but no weight was recorded (Fig. 9b). The relationship between length and weight was:

$$ W=0.00004569\ast {TL}^{2.753} $$

The parameters *a* (*t* = 6.01, *p* = 3.44e^-09^) and *b (t* = 69.20, *p* < 2e^-16^) were both significant (Fig. 9a).

Coxa 4 varied in length between 6.1 and 12.9 mm for females and 1.9–10.3 mm for juveniles. The relationship between total length and coxa 4 (*t* = 132.281, *p* < 2e^-16^, *r*^2^ adjusted = 0.9694) followed a linear relationship (Fig. 9b):

$$ TL=2.46262\ \left(\mathrm{standard}\ \mathrm{error}\pm 0.36585\right)+6.19965\ \left(\mathrm{standard}\ \mathrm{error}\pm 0.04687\right)\ast coxa4 $$

The relationship between total length and depth appeared to follow a sigmoidal relationship for juveniles (Fig. 9c). Total length remains constant with depth (33.7 ± 19.6 mm) until ~6500 m before it begins to increase. Around 7200 m, the relationship between total length of juveniles begins to increase rapidly and then reaches an asymptote by ~7700 m (59.9 ± 8.2 mm). The inflection point of the sigmoidal relationship is at ~7300 m, and no juveniles were smaller than 35 mm beyond this depth. There was no relationship between total length and depth in females (Spearman correlation: rho = 0.05, *p* = 0.3235; Fig. 9d).

## Discussion

This study described a scavenging amphipod endemic to the Peru-Chile Trench, *Eurythenes atacamensis* sp. nov., by applying an integrative taxonomic approach. Analysis of the Atacamex and RV Sonne SO261 Expeditions specimens expanded the bathymetric range of *E. atacamensis* sp. nov. from only hadal to include abyssal depths, confirmation of ontogenetic vertical stratification across the trench axis, and revealed differing size-to-depth trends between juveniles and females.

**Placement within *Euythenes***. *Eurythenes atacamensis* sp. nov. represents a unique lineage within *Eurythenes*. The two mtDNA topologies supported that the *Eurythenes* sp. ‘PCT hadal’ recovered from SO209 Expedition are *E. atacamensis* sp. nov. (Fig. 7; Ritchie et al. 2015; Eustace et al. 2016). While comparative sequences from the SOUTHTOW, SIO BI72–20, and ATIE specimens were not available, the combination of the morphological characterization, photographs, and sampling locations provide sufficient evidence to conclude they were *E. atacamensis* sp. nov. (Hessler et al. 1978; Ingram and Hessler 1987; Thurston et al. 2002). Notably, this species is not part of the *gryllus* complex and basally rooted within the presented phylogenies, more closely related to *E. thurstoni* and *Eurythenes* sp. ‘Eg9’ (France and Kocher 1992; Havermans et al. 2013). Morphologically, *E. atacamensis* sp. nov. possesses distinguishable and non-cryptic characteristics (d’Udekem d’Acoz and Havermans 2015), specifically the short palm of gnathopod 1 and chelate palm of gnathopod 2 (Fig. 4). *Eurythenes thurstoni* is the only other known *Eurythenes* species with a chelate gnathopod 2 (Stoddart and Lowry 2004). Evidence suggests that among gammaridean amphipods, the gnathopods serve a range of functions, including feeding (Klages and Gutt 1990), grooming (Holmquist 1985), and reproduction (Borowsky 1984). However, the current dearth of understanding regarding the functional significance of gnathopod morphological differences in *Eurythenes* amphipods precludes the assignment of any particular selection pressure as the driver of this divergence.

**Bathymetric and geographic distribution**. Based on historical expeditions, *E. atacamensis* sp. nov. was considered restricted to hadal depths, with a total range of 1901 m (Eustace et al. 2016). These two expeditions have expanded the known bathymetric range of *E. atacamensis* sp. nov. to 3099 m, showing it is not restricted solely to hadal depths. Rather, *E. atacamensis* sp. nov. spans the abyssal-hadal transition zone and extends to the deepest point in the Peru-Chile Trench (Fig. 7). Eurybathic distribution is common within *Eurythenes*, specifically *E. gryllus*, *E. magellanicus*, *E. maldoror*, and *E. sigmiferus* have ranges spanning over 2500 m (Escobar-Briones et al. 2010; d’Udekem d’Acoz and Havermans 2015; Lacey et al. 2016). As with geographic distributions, the known bathymetric range of *Eurythenes* species is likely to continue widening with expanded global sampling efforts (Havermans 2016).

The latitudinal distribution of *E. atacamensis* sp. nov. spans the entire Peru-Chile Trench, with the presence at the Milne-Edwards Trench (northern sector; Eustace et al. 2016) and the Atacama Trench (southern sector; Fig. 1a; Hessler et al. 1978; Ingram and Hessler 1987; Thurston et al. 2002; Eustace et al. 2016). While this could be the full extent of their distribution, it remains outstanding whether the distribution extends west to the neighbouring abyssal plains. As this study found juveniles as shallow as 4974 m at the Atacama Trench, it is curious that no specimens were previously collected from abyssal depths (4602 and 5329 m) in the Milne-Edwards Trench (Eustace et al. 2016). This could be a false absence. Another possibility is the abyssal absence in the Milne-Edwards Trench reflects distributional differences in response to the distinctive environmental and surface productivity conditions of each trench sector. For instance, the Milne-Edwards Trench is considered sediment-starved with highly productive year-round upwelling, while the Atacama Trench has high sediment loads with seasonal upwelling (Montecino and Lange 2009; Geersen et al. 2018; Geersen 2019). Additionally, the Nazca Ridge partitioning the two sectors of the Peru-Chile Trench is ~4000 m deep (Fig. 1a; Hampel et al. 2004). It remains outstanding whether this is a barrier to *E. atacamensis* sp. nov. Future research investigating population connectivity across the Nazca Ridge and the role of environmental and surface productivity differences between the two sectors would enhance the interpretation of their population structure and distribution ecology.

Along with *E. atacamensis* sp. nov., *E. magellanicus* and *E. maldoror* co-occurred at the shallowest station of the RV Sonne SO261 Expedition (4974 m). *Eurythenes magellanicus* is known from the Milne-Edwards Trench (Eustace et al. 2016), and this study expands its range southward into the Atacama Trench. Further, this is the first account of *E. maldoror* in the Peru-Chile Trench, expanding its distribution to the eastern South Pacific Ocean (Havermans 2016; Weston et al. 2021). Surprisingly, *Eurythenes* sp. ‘PCT abyssal’ was not recovered, as previously found at the Milne-Edwards Trench (Eustace et al. 2016). This may indicate that *Eurythenes* sp. ‘PCT abyssal’ is restricted to the Milne-Edwards Trench. Together, the presence of *E. magellanicus*, *E. maldoror*, and *E. atacamensis* sp. nov. highlights the complexity of the patchwork geographic and bathymetric distributions within *Eurythenes*.

**Population structure and life history**. Ontogenetic vertical stratification was evident with the RV Sonne SO261 specimens, whereby juveniles dominated the upper depths (<6714 m), and females were dominant at the deeper depths (>7139 m; Fig. 8). Ontogenetic vertical stratification by *E. atacamensis* sp. nov. is not novel to *Eurythenes* or the Peru-Chile Trench (Eustace et al. 2016). Similar instances have been documented in other abundant hadal scavenging amphipods, including *Bathycallisoma schellenbergi* (Birstein and Vinogradov, 1958) from the Kermadec and New Hebrides trenches (Lacey et al. 2018), *Hirondellea dubia* Dahl, 1959 from the Tonga and Kermadec trenches (Blankenship et al. 2006; Lacey et al. 2018; Wilson et al. 2018), and *Hirondellea gigas* (Birstein & Vinogradov, 1955) from the Izu-Bonin Trench (Eustace et al. 2013). This demographic trend was consistent with the SO209 expedition, indicating that the ontogenetic vertical stratification pattern is constrained by depth in the Peru-Chile Trench and not confounded by latitude. Lacey et al. (2018) proposed that ontogenetic vertical stratification is an ecological strategy to reduce competition for food resources and alleviate pressure-induced physiological and metabolic limitations. The bathymetric trends in the size of *E. atacamensis* sp. nov. support this hypothesis, and further reveal this strategy is applied differently by females and juveniles.

Based on this dataset and the body of literature on *Eurythenes* biology, it is plausible to visualize the following population dynamic for *E. atacamensis* sp. nov. in the Atacama Trench. Here, the early-stage juveniles are small (<30 mm; Fig. 9c) and have not built-up wax esters and lipid reserves (Bühring and Christiansen 2001). Thus, they may be constrained to locating food over a small area (Hargrave et al. 1994) at the shallower depths. The trade-offs to living at depths with lower metabolic pressures are food resources at a lower concentration (Danovaro et al. 2003; Ichino et al. 2015; Glud et al. 2021) and predation risks (Havermans and Smetacek 2018) from fauna such as cusk eels, snailfish, or penaeid decapods (Wenzhöfer 2019). As the juveniles grow to a later stage (Fig. 9c), their extra lipid reserves and larger body size perhaps allow them to descend to depths beyond predatory species (Wilson and Ahyong 2015; Linley et al. 2016), and then they exploit the higher concentration of phytodetritus and organic carbon to continue developing towards maturity (Danovaro et al. 2003; Ichino et al. 2015; Lacey et al. 2018; Glud et al. 2021). The females have the lipid reserves, metabolic capacity, and strong swimming ability (Havermans 2016) to expand their horizontal and vertical ranges (Fig. 9d; Hargrave et al. 1994) across the trench axis.

While the bathymetric trend of juveniles and females across hadal depths can be rationalized, it remains less clear why small stage juveniles were found ~1500 m shallower than the shallowest females (Fig. 8). More questionably, how do the small stage juveniles arrive at abyssal depths? Ovigerous females are presumed to stop feeding to prevent expulsion of the brood (Bregazzi 1972; Christiansen et al. 1990; Johnson et al. 2001; Lacey et al. 2018) and are thus systematically excluded from the baited traps. Several hypotheses, constrained by the lack of behaviour and bathymetric evidence of ovigerous females, may explain the presence of abyssal *E. atacamensis* sp. nov. juveniles. Previously work has postulated that the Atacama Trench population receives continuous recruitment from abyssal depths (Thurston et al. 2002). However, this is a less plausible scenario, given that adults have not been found shallower than 6103 m (Eustace et al. 2016), high abundance at hadal depths, and the nearby abyssal plains have not been sampled. Another potential explanation is that females release their hatchlings at shallow hadal depths. The newly hatched juveniles, with functional mouthparts and developed pleopods (Thurston and Bett 1995), then migrate to even shallower, abyssal depths. While there are metabolic benefits to migrate shallower, this transit to shallower depths with low lipid reserves is challenging to reconcile. A more complex hypothesis is that ovigerous females migrate and release their brood between the abyssal and shallow hadal depths (~4900–6500 m), and then those females die shortly after. *Eurythenes* are assumed to be iteroparous (Ingram and Hessler 1987) and have an extreme K-selected to A-selected life history due to nutrient limitations (Sainte-Marie 1991). Yet, none of the recovered *E. atacamensis* sp. nov. females had fully setose oostegites, which would suggest an interim resting stage between broods. This lack of fully mature females is consistent with Eustace et al. (2016) and Thurston et al. (2002). Thurston et al. (2002) suggested that the eutrophic environment of the Atacama Trench would release them from an extreme K-selected strategy. Thus, with the high level of resources in the Atacama Trench, *E. atacamensis* sp. nov. may fall more towards semelparity on the semelparous-iteroparous continuum (Varpe and Ejsmond 2018). *Hirondellea thurstoni*, also a hadal endemic in the Atacama Trench, is considered to display a semelparous life history strategy (Perrone et al. 2002). Another feature that is challenging to reconcile is the lack of males. This skewed sex ratio was similarly found by Thurston et al. (2002) and Eustace et al. (2016), which indicates that males are either not attracted to the bait or not present. Unlike ovigerous females, no evidence suggests a lack of attraction to bait. While the lack of males is curious, the evidence is insufficient to speculate on their absence. Confirmation of any of these hypotheses on the abyssal presence of juveniles and more broadly the life history strategy of the *E. atacamensis* sp. nov. warrants further investigations. Future work would benefit from additional sampling to assess seasonal population dynamics and more detailed instar analysis.

**Significance for *Eurythenes***. *Eurythenes atacamensis* sp. nov. represents a key addition to *Eurythenes*, one of the most intensely studied genera of deep ocean Amphipoda. This species represents a unique lineage with its eurybathic distribution across the abyssal and hadal depths of the eutrophic Peru-Chile Trench. This study highlights the importance of systematic sampling expeditions to resolve the geographic and bathymetric range of a species more fully. Further research of *Eurythenes* should continue to apply an integrative taxonomic approach and work towards a fuller understanding of their life histories. Together, this will ultimately lead to increased understanding of the biogeographic ranges of these key deep-ocean fauna and the eco-evolutionary drivers of speciation in the world’s largest ecosystem.

## Declarations

### Sampling and field studies

All necessary permits for sampling in Chile's Exclusive Economic Zone were obtained by the authors from the Servicio Hidrográfico y Oceanográfico de la Armada de Chile. The study is compliant with CBD and Nagoya protocols.

### Data availability

All genetic sequences have been deposited into NCBI GenBank under accession numbers MW042878–MW042884, MW290039 for 16S sequences and MW048992–MW048996, MW288146 for COI sequences.
